# Systematic screening for novel, serologically reactive Hepatitis E Virus epitopes

**DOI:** 10.1186/1743-422X-9-28

**Published:** 2012-01-23

**Authors:** Andreas Osterman, Maria Guadalupe Vizoso Pinto, Rudolf Haase, Hans Nitschko, Simone Jäger, Michaela Sander, Manfred Motz, Ulrich Mohn, Armin Baiker

**Affiliations:** 1 Max von Pettenkofer-Institute, Department of Virology, Ludwig-Maximilians-University Munich, Pettenkoferstrasse 9a, 80336 Munich, Germany; 2 Mikrogen GmbH, Floriansbogen 2, 82061 Neuried, Germany; 3 Bavarian Health and Food Safety Authority, Veterinaerstrasse 2, 85764 Oberschleissheim, Germany

## Abstract

**Background:**

The National Institutes of Health classified Hepatitis E as an emerging disease since Hepatitis E Virus (HEV) is the major cause of acute hepatitis in developing countries. Interestingly, an increasing number of sporadic cases of HEV infections are described in industrialized countries as zoonosis from domestic livestock. Despite the increasing relevance of this pathogen in clinical virology, commercial antibody assays are mainly based on fragments of HEV open reading frame (ORF) 2 and ORF3. The largest ORF1 (poly-)protein, however, is not part of current testing formats.

**Methods:**

From a synthesized full length HEV genotype 1 cDNA-bank we constructed a complete HEV gene library consisting of 15 respective HEV ORF domains. After bacterial expression and purification of nine recombinant HEV proteins under denaturating conditions serum profiling experiments using 55 sera from patients with known infection status were performed in microarray format. SPSS software assessed the antigenic potential of these nine ORF domains in comparison to seven commercial HEV antigens (genotype 1 and 3) by performing receiver operator characteristics, logistic regression and correlation analysis.

**Results:**

HEV antigens produced with our method for serum profiling experiments exhibit the same quality and characteristics as commercial antigens. Serum profiling experiments detected Y, V and X domains as ORF1-antigens with potentially comparable diagnostic significance as the well established epitopes of ORF2 and ORF3. However no obvious additional increase in sensitivity or specificity was achieved in diagnostic testing as revealed by bioinformatic analysis. Additionally we found that the C-terminal domain of the potential transmembrane protein ORF3 is responsible for IgG and IgM seroreactivity. Data suggest that there might be a genotype specific seroreactivity of homologous ORF2-antigens.

**Conclusions:**

The diagnostic value of identified ORF1 epitopes might not necessarily improve sensitivity and specificity, but broaden the overall quality of existing test systems. ORF2 and ORF3-antigens are still commonly used in diagnostic assays and possibly hold the potential to serologically differentiate between genotype 1 and 3 infections. Our systematic approach is a suitable method to investigate HEV domains for their serologic antigenicity. Epitope screening of native viral domains could be a preferable tool in developing new serologic test components.

## Background

Four different genotypes of Hepatitis E Virus (HEV) are known to infect mammals. The majority of HEV epidemics in Asia, Africa and Latin America (Mexico) have been caused by genotype 1 and 2 [1]. In these endemic regions the virus is usually transmitted fecal-orally and spread through contamination of drinking water often related to flood and heavy rainfall [2]. However HEV is also endemic to industrialized countries of Europe, Asia and the U.S., where the number of sporadic cases of hepatitis E of genotype 3 and 4 has increased in recent years [3]. While genotypes 1 and 2 seem to be restricted to humans, genotypes 3 and 4 have a high prevalence in pig populations world-wide. Hepatitis E is now regarded as a zoonotic disease and pigs and most likely other animal species are reservoirs [4]. In addition to genotypes 1-4, novel genotypes have been detected in wild boars from Japan [5] and in rats from Germany [6].

Generally, hepatitis E is a self-limiting disease with low mortality. However severe courses of the disease with acute liver failure have been reported during pregnancy [7] and in patients with liver cirrhosis [8]. So far prolonged courses have been registered only in organ transplant recipients [9] and patients suffering from leukemia [10].

**Figure 1:**
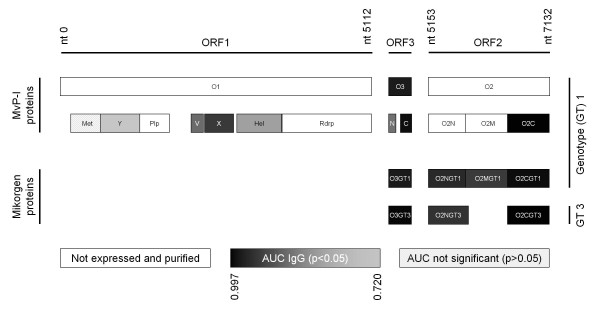
**Overview of investigated epitopes of the HEV genome**. Schematic overview of the HEV genome. Bars indicating defined domains. Filling reflects IgG seroreactivity and grey scale correlates with area under the curve (AUG) (see Table 1). Nucleotide (nt) numbers indicating position in the synthesized sequence.

HEV is a non-enveloped, single stranded (+) RNA virus classified in the Hepeviridae family [11] with a 7.2 kb HEV genome encoding for three partly overlapping open reading frames (ORF) and a capped 5' and polyadenylated 3' end. ORF1 comprises several putative functional domains [12] (Figure 1): A methyltransferase (Met) with subsequent Y domain; a papain-like cysteine protease (Plp), which over a proline-rich variable (V) region is connected to the so-called X domain; a helicase (Hel) and a RNA-dependent RNA polymerase (RdRp). ORF2 encodes for the viral capsid protein, which contains dominant antigenic determinants [13] and neutralizing epitopes [14]. ORF 3 encodes a small phosphoprotein [15] of controversially discussed function that seems to be essential for in vivo infectivity [16,17].

Currently different synthetic peptides and recombinant antigens of ORF2 and 3 of genotypes 1, 2 and 3 are used for commercially available serological tests (enzyme linked immunosorbent assay (ELISA), line-immuno-assay (LIA)) and the most relevant related publications are limited to the description of these antigens. Studies characterizing immunogenic peptides in regions of all three ORFs of porcine [18] and human [19,20] hepatitis E virus are limited to a non-systematic approach or have not yet transferred their results to the field of diagnostic routine testing.

In this work we present a systematic strategy for screening of the entire proteome of HEV for the identification of serologically reactive HEV antigens based on recombinant, bacterially expressed and purified HEV proteins. For this purpose, fifteen potentially immunogenic HEV antigens of ORFs 1, 2 and 3 were recombinatorially cloned into bacterial expression vectors of which nine were subsequently expressed and purified. Their immunogenicity was evaluated with pre-characterized blood samples on a microarray format in comparison to seven recombinant antigens already in use in a commercially available anti-HEV diagnostic test-system. Analyzed data show that seven of those nine HEV proteins were putative serologic markers of HEV infections. Statistical analysis of measured signal intensities could confirm well known epitopes, identify immunogenic subdomains and characterize newly described antigens and their possible suitability in commercial assay formats.

## Methods

### Recombinatorial cloning of a complete HEV library

**Figure 2:**
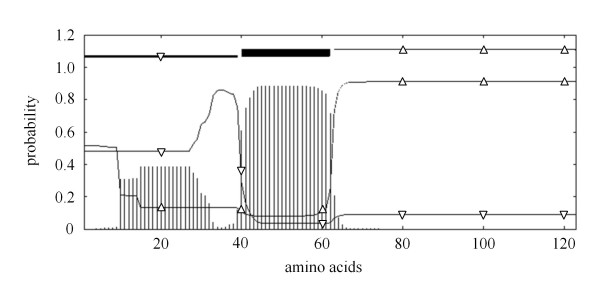
**Prediction of transmembrane helices in HEV ORF3 protein**. TMHMM posterior probabilities for sequence; vertical bars: transmembrane; triangle down: inside; triangle up: outside.

The genotype 1 HEV genome synthesized by Geneart (Regensburg, Germany) served as template for subcloning of a complete, Gateway^® ^compatible pENTR207 (Invitrogen, Germany) HEV library consisting of 15 HEV ORFs and ORF fragments: ORF1, ORF2 and ORF3 full length, additionally eight functional ORF1 domains [13] (amino acid positions as defined by [genebank:L08816]) and three ORF2 fragments (amino acid positions analogue to ORF2 sequence [genebank:L08816]; O2A: 1-218; O2B: 206-451; O2C: 432-660). Since a transmembrane domain of ORF3 was predicted by the TMHMM online tool http://www.cbs.dtu.dk/services/TMHMM[21] we additionally subcloned an N-terminal and C-terminal ORF3 fragment (amino acid positions analogue to ORF3 sequence [genebank:L08816]; O3N: 1-39; O3C: 63-123) (Figure 2). Recombinatorial cloning of the HEV ORFs was performed as described recently [22].

### Expression and purification of His-tagged HEV antigens

**Figure 3:**
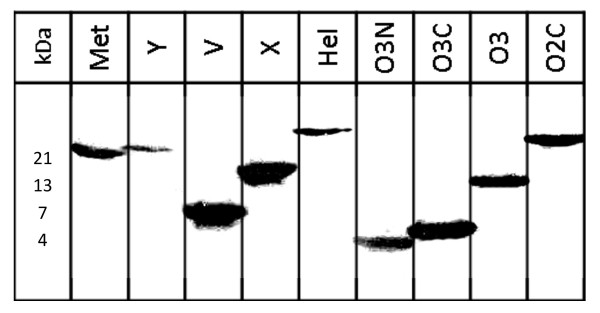
**Western Blot of recombinant HEV proteins**. Bacterially expressed and purified N-terminally His-tagged HEV proteins detected by monoclonal mouse anti RGS-His antibody. Protein mass in kDa as expected.

Bacterial expression vectors pETG-A-His-N-HEV-ORFs were transformed into *E. coli *Rosetta (DE3). 400 ml LB-medium main cultures supplemented with ampicillin were incubated at 30°C, inducted with 1 mM Isopropyl β-D-1-thiogalactopyranoside and grown for 3 h at 30°C. After centrifugation bacterial pellets were resuspended and incubated in ice-cold lysis buffer (10% glycerol, 20 mM Tris-HCl, 0.5 M NaCl, 5 mM Imidazole, pH 7.9, supplemented with DNAse, RNAse, proteinase inhibitors, and lysozym) and homogenized using the MagnaLyser (Roche Applied Science, Germany) according to manufacture's instructions. After sonication, protein inclusion bodies were pelleted by centrifugation, resuspended, sonicated and incubated in binding buffer (0.5 M NaCl, 5 mM Imidazol, 20 mM Tris-HCl, 8 M urea, pH 7.9). After centrifugation His-tagged proteins from supernatants were purified using HisBind Columns (Novagen, Germany) according to the steps described by the manufacture using buffers with an Imidazole gradient up to 250 mM for elution. Expression and degree of purification of recombinant N-terminally His-tagged HEV proteins were analyzed by SDS/PAGE followed by Coomassie staining and verified by Western blotting using monoclonal mouse anti RGS-His antibody (Qiagen, Germany) (Figure 3). Purified proteins were stored at -20°C.

### Antigen-immunogenicity evaluation experiments in microarray format

For immunogenic evaluation, nine small scale purified HEV antigens were spotted onto nitrocellulose microarrays (Max von Pettenkofer-Institute (MvP-I) antigens). Additionally incubation and conjugate control spots as well as seven purified recombinant proteins already included in a commercially available anti-HEV diagnostic test-system (recomLine HEV, Mikrogen, Germany) were spotted on the same membranes: the N- (O2NGT1, O2NGT3), C- terminal (O2CGT1, O2CGT3) (each of genotype (GT) 1 and 3) and the middle part (O2MGT1) (of genotype 1) of ORF2 as well as the complete ORF3 (each of genotype 1 and 3: O3GT1, O3GT3) (Figure 1). Publication of the exact locations and amino acid compositions of the used commercially produced antigens was not possible as this information was considered proprietary. The microarrays (*recom*Dot, Mikrogen, Germany) were processed according to manufacturer's instructions. Arrays were incubated with 2 ml diluted serum (1:40 in *recom*Dot buffer, Mikrogen, Germany), washed with *recom*Dot washing buffer (Mikrogen, Germany) and for detection of specifically bound antibodies with anti-human IgG- and anti-human IgM-peroxidase labeled conjugates (Seramun, Germany). Visualization of immune complexes was achieved by tetramethylbenzidine substrate (Seramun, Germany). After automated scanning, the quantification of specific signals was performed by digitalization of grey tones with the *recom*Dot Scan software (*recom*Dot system, Mikrogen, Germany).

### Blood samples used for evaluation

**Figure 4:**
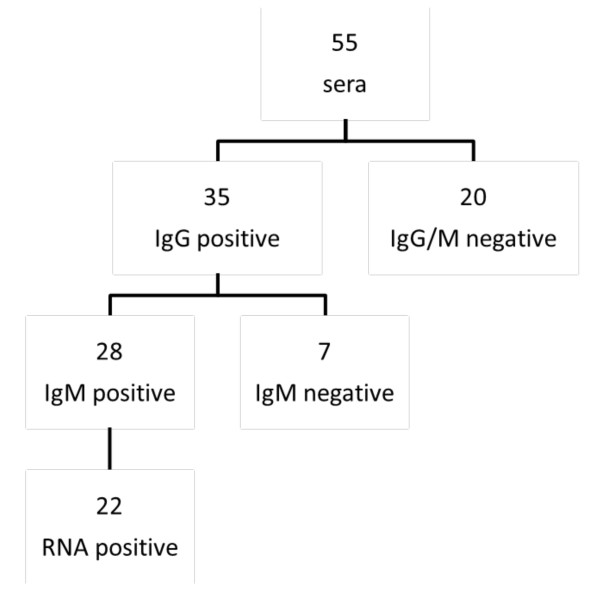
**Overview serum samples**. Pre-characterization by HEV *recom*LINE (Mirkogen, Germany), HEV ELISA (Genelabs Diagnostics, Singapore) and in-house PCR.

All samples were pre-characterized for the presence of HEV-IgG and IgM by *recom*Line HEV (Mikrogen, Germany) and IgG negative sera additionally by HEV ELISA (Genelabs Diagnostics, Singapore) with consistent results. In order to identify immunogenic HEV antigens, the microarrays were probed with blood samples from clinically healthy blood donors (n = 20, defined as IgG and IgM negative) and from patients suffering from an acute HEV infection including follow up samples (N = 35). In the group of Hepatitis E patients, 35 were defined as IgG positive and 28 IgM positive of which 22 had detectable HEV RNA (Figure 4). To test patient samples for the presence of HEV RNA, a routine diagnostic PCR amplifying a fragment of the conserved 5' non-coding-region was used. A few amplicons were sequenced with a CEQ™ 8,800 Genetic Analysis System (Beckman Coulter Inc., Fullerton CA, USA) and sequences compared with Genebank entries, using the BLAST application [23]. Sequence analysis of 10 sera showed three genotype 1, two genotype 3 and five genotype 4 infections, respectively.

### Statistic analysis

All statistical tests were performed by the SPSS 18.0.0 software for Windows. Sensitivity and specificity were calculated with binary logistic regression methods. Additionally performance of antigens was evaluated by comparing Receiver Operator Characteristics (ROC) curves and calculated area under the curves (AUCs). A bivariate correlation (Pearson) > 0.8 was defined as a high correlation. Results were accepted as significant if the *p*-value was < 0.05.

For binary regression analysis inclusion and exclusion criteria for variables are based on Pearson correlation coefficients. In this study this method can be explained as followed: The backward method starts with an assay consisting of all available antigens. If an antigen does not have an explanatory value which is significant for the assay the antigen is removed in the next step. This method discontinues when no further antigen can be removed from the set of antigens without significant loss of the explanatory value of the assay. Analogously the forward method starts with a single antigen which has the highest explanatory value of all available antigens. In the following step only an antigen which increases the explanatory value of the hole assay is added to the set of antigens. The forward method discontinues when the addition of a further antigen to the assay does not improve the explanatory value of current antigens [24].

## Results

### Serum profiling experiments of pre-characterized sera in microarray format

9 out of 15 HEV proteins (60%) could be detected in bacterial lysates and purification of all 9 N-terminally His-tagged proteins was verified by western blot analysis using monoclonal mouse anti RGS-His antibody, namely: Met, Y, V, X, Hel, O3N, O3C, O3, O2C (Figure 3). In microarray format the proteins Y, V, X, Hel, O3N, O3C, O3, O2C differentiated significantly between IgG positive and negative sera (*p *< 0.05) (Figure 1). Met, Y, V, X, O3N, O3C, O3, O2C significantly discriminated between sera of IgM positive patients and negative control patients (*p *< 0.05).

### Combination of antigens for optimal test performance

The best possible antigen combination for the optimal test performance was calculated using forward and backward binary regression methods comparing negative control sera with all sera positive for IgG and IgM, respectively. When choosing from all 16 antigens in IgG detection using the backwards method, it discontinued with Y and O2CGT3, which were left over in the last step. Consequently, the removal of one of these two antigens would change the explanatory value of the assay significantly. Similar, when choosing only from the seven "Mikrogen antigens" the same method discontinued with O2NGT1 and O2CGT3. Comparing both antigen combinations, binary regression analysis showed that there is no statistically significant difference between both combinations. In IgM detection a similar result was obtained: Choosing from 16 antigens the backwards method reveals V, O2CGT3 and O3GT3 to be the best combination. If restricted to the seven "Mikrogen antigens" only, O2NGT3 was set instead of V, but binary regression analysis could not determine any statistically significant difference between both antigen combinations. Further calculation of a forward binary regression method used to obtain the best antigen combination choosing from all 16 antigens resulted in O2CGT3 in combination with O2NGT1 for IgG detection. This means that the addition of any other antigen would not increase the explanatory value of the assay consisting of these two antigens. O2CGT1 alone is sufficient as diagnostic marker for IgM detection, as calculated by the forward regression method.

### Correlation of serologic reactivities of ORF2 and ORF3 antigens (Tables 2 and 3)

Analysis of bivariate correlations between ORF3 subdomains, homologous GT1 ORF3 and homologous GT1 ORF2 antigens in IgG- and IgM seroreactivities are shown in Tables 2 and 3. Among ORF3 antigens only low and moderate correlations of O3N with other ORF3 antigens (O3C, O3 and O3GT1) was found for IgG- and IgM-seroreactivities. In contrast O3C seroreactivities exhibited a high correlation with O3 and O3GT1 in IgG and IgM. O3 and O3GT1 were also found to correlate very highly in IgG and IgM. Analogue O2C and O2CGT1 showed a high correlation of IgG and IgM seroreactivities.

### Sensitivities and specificities of HEV antigens (Table 1)

Table 1 shows specificity and sensitivity of IgG and IgM of all tested antigens. These results were obtained by calculation of binary regression analysis and AUC of ROC. Since regression analysis of Met in IgG- and Hel in IgM-testing did not generate significant (*p *> 0.05) results, these two antigens were not pursued in subsequent analysis.

**Table 1:** Specificity, Sensitivity and area under the curve of Hepatitis E Virus antigens

origin of antigen	Name	Specificity IgG (%)	Sensitivity IgG (%)	AUC IgG (ROC)	Specificity IgM (%)	Sensitivity IgM (%)	AUC IgM (ROC)
MvP-I antigens	Met	5.0^1^	88.6^1^		65,0	78,6	0,727
	Y	50,0	80,0	0,720	55,0	82,1	0,735
	V	65,0	77,1	0,826	80,0	75,0	0,877
	X	75,0	77,1	0,899	70,0	78,6	0,823
	Hel	55,0	80,0	0,761	50.0^1^	85.7^1^	
	O3N	65,0	82,9	0,819	40,0	75,0	0,692
	O3C	95,0	94,3	0,974	95,0	89,3	0,941
	O3	90,0	94,3	0,950	85,0	78,6	0,920
	O2C	95,0	97,1	0,996	100,0	100,0	1,000

Mikrogen antigens	O3GT1	85,0	88,6	0,951	95,0	89,3	0,964
	O3GT3	90,0	94,3	0,986	95,0	96,4	0,996
	O2NGT1	85,0	82,9	0,926	100,0	92,9	0,979
	O2NGT3	90,0	80,0	0,910	90,0	89,3	0,938
	O2MGT1	85,0	80,0	0,898	90,0	75,0	0,896
	O2CGT1	90,0	94,3	0,977	100,0	100,0	1,000
	O2CGT3	100,0	97,1	0,997	90,0	89,3	0,984

^1^*P *> 0.05, *AUC*: Area under the curve, *ROC*: Receiver Operator Characteristic

**Table 2:** Correlation coefficients of ORF2 and ORF3 antigen IgG-reactivities

	O3N	O3C	O3	O3GT1	O2C	O2CGT1
O3N	1^1^	0.715	0.734	0.605		
O3C		1	0.982	0.896		
O3			1	0.915		
O3GT1				1		

O2C					1	0.943
O2CGT1						1

^1^Numbers indicate Pearson correlation coefficient

**Table 3:** Correlation coefficients of ORF2 and ORF3 antigen IgM-reactivities

	O3N	O3C	O3	O3GT1	O2C	O2CGT1
O3N	1^1^	0.327	0.449	0.336		
O3C		1	0.932	0.897		
O3			1	0.903		
O3GT1				1		

O2C					1	0.900
O2CGT1						1

^1^Numbers indicate Pearson correlation coefficient

Among all antigens Y performed with the lowest specificity in both IgG and IgM detection. Simultaneously the Y protein achieved the highest sensitivity rates among ORF1 proteins in IgG and IgM detection. V performed with the lowest sensitivity rates among all 16 antigens in IgG as well as IgM detection, but simultaneously highest ORF1 specificity in IgM detection and generated the largest AUC in IgM ROC analysis among ORF1 antigens. The largest AUC in IgG ROC analysis among ORF1 antigens was generated by X protein with a value of 0.899, which is notably higher than the AUC generated by O2MGT1 (0.898) and O3N (0.819) in this antibody class.

The best performance in IgG detection among all 16 antigens was achieved by O2CGT3 with a specificity of 100%, sensitivity of 97.1% and AUC of 0.997. The lowest results in IgG and IgM detection among ORF2 proteins were detected in O2MGT1 antigen specificities, sensitivities and AUCs. Optimal results regarding IgM detection were accomplished by O2CGT1 and O2C with specificities and sensitivities of 100% resulting in an AUC of 1. O2NGT1 had an IgM specificity of 100%.

O3N shows clearly the lowest diagnostic performance of all ORF3 antigens in both IgG and IgM detection. Among ORF3 proteins best values in IgG detection were accomplished by O3GT3. Only O3C showed with 95.0% a higher specificity value than O3GT1. In IgM detection O3GT3 scored highest in specificity, sensitivity and AUC among ORF3 antigens.

### Genotype-specific reactivity of O2NGT1/3 and O2CGT1/3 antigens (Table 4)

**Table 4:** Genotype-specific reactivity of homologous HEV genotype 1 and 3 antigens

Serum	Antigen					
**HEV genotype**	**O2N GT3/GT1^1^**		**O2C GT3/GT1^1^**		**O3 GT3/GT1^1^**	
	**IgG**	**IgM**	**IgG**	**IgM**	**IgG**	**IgM**

1	0,723	0,572	0,973	0,556	0,881	0,125
	0,927	0,254	0,899	0,970	0,449	0,074
	0,923	0,689	0,935	0,840	0,923	0,434

3	**2,048**	**2,163**	**1,019**	**1,096**	0,668	0,571
	**16,387**	**22,586**	**1,074**	**1,335**	0,929	0,522

4	**3,270**	**1,414**	0,706	0,016	0,322	0,131
	**1,281**	**1,442**	0,842	0,979	0,753	0,181
	**3,448**	**1,737**	0,971	0,271	0,390	0,145
	**1,220**	0,570	**1,055**	0,848	0,896	0,330
	**1,064**	0,907	**1,013**	0,921	0,886	0,292

^1^ratios: signal intensities of genotype 3/genotype 1 antigen

bold: signal intensity genotype 3 > genotype 1

To assess the potential of homologous genotype 1 and 3 antigens from ORF2 and 3 to discriminate between sera from different genotypes the quotient of measured signal intensities was calculated. Higher O2N and O2C IgG- as well as IgM-signal intensities were measured when genotype 1 antigen was incubated with genotype 1 sera (n = 3) and genotype 3 antigen with genotype 3 sera (n = 2). No such correlation was found for O3 genotype 1 and 3, where reactivity of genotype 1 antigen was always higher than of genotype 3 antigen. When genotype 1 and 3 antigens were incubated with genotype 4 sera (n = 5) the results were inconsistent either between O2N and O2C (3/5) or between IgG and IgM (2/5).

## Discussion

Over the last years increasing numbers of autochthonous cases, zoonotic spread and chronic infections has led to a better understanding of viral hepatitis E [25]. The prevalence of anti-HEV in Central Europe may reach up to 15% [26]. It is unclear whether this high prevalence is caused by a high number of undiagnosed cases of subclinical HEV infections or by a high false positive rate of unreliable serologic HEV antibody assays. Despite the increasing epidemiological and clinical relevance of the HEV and demanding research needs [3], available serological tests are limited to the detection of antibodies against so-called "traditional" antigens ORF2 and ORF3 [1].

In this study we describe a novel seroreactivity for ORF1 domains and extensively characterized it for their diagnostic potential in direct comparison to commercial available ORF2 and ORF3 antigens. The sequence of the potential ORF1 antigen was determined by the probably most native structure namely defined functional domains. Other studies of putative ORF1 epitopes are based on computer analysis of hydrophobicity and secondary structure [18,20] or overlapping decamers [19]. A common disadvantage of these artificially predicted epitopes is the presence of only linear epitopes and therefore conformational antigens might be missed [19]. ORF1 epitopes found in earlier studies were within the regions of Met [18,19], Plp [19], V [19], × [18,19], Hel [19], Rdrp [18-20]. According to Zhao et al. the major antigenic epitopes of HEV are located in the ORF2 and ORF3. Four ORF1 antigens were excluded early in the study after initial experiments showed inadequate immunoreactivities of ORF1 antigens [18]. Kaur et al. used two serum pools to screen for reactive ORF1 polypeptides but did not investigate the diagnostic impact with sensitivity and specificity levels of the distinct ORF1 proteins [19]. Overall these data revealed that the diagnostic impact of ORF1 antigens is low. Only Qi et al. could show that the inclusion of an Rdrp epitope in a serologic assay increases the test performance for an unknown reason but did not focus on the performance of other ORF1 epitopes [19]. In our study we also calculated if the additional presence of an ORF1 antigen could complement ORF2 and ORF3 antigens in a diagnostic test. It became clear that the two ORF1 antigens included in the diagnostic test according to backwards regression analysis (Y in IgG detection and V in IgM detection) could be exchanged by antigens already used in commercially test without significant change of the diagnostic performance.

For the first time we are able to describe immunogenic properties of the Y protein, a protein with homologies to non-structural proteins of the Rubella virus and beet necrotic yellow vein virus [12]. This finding is in line with results from all other ORF1 regions and completes the list now consisting of all ORF1 domains exhibiting a moderate immunogenicity. This is easily explained by the fact that all parts of the ORF1 polyprotein are expressed in the same amount in the cytoplasm of infected cells, processed and presented to the immune system equally. Nevertheless, Y shows in our study the highest IgG and IgM sensitivities among all investigated ORF1 domains.

Within the X domain we measured higher sensitivities and specificities compared to a peptide from this region detected by Zhao et al. [19] who investigated its seroreactivity exclusively in swine. In our study values from the X-protein in human HEV diagnostics even showed a better performance than commercial O2MGT1 in detection of IgG and IgM. Interestingly, it was shown, that X-protein impacts viral pathogenicity of a murine Corona Virus (MHV A59) [27,28].

In contrast to the predictions of Zhao et al. [18] and Kaur et al. [19] we could not show any antigenic properties within the methyltransferase domain. Nevertheless, this does not necessarily exclude the possibility of a diagnostic value of ORF1 antigens under different conditions like for example investigations in endemic regions with a larger patient population. By changing the methodical approach of protein expression, further studies should investigate the seroreactivity of the remaining ORF1 domains not expressed in this study as for example the polymerase domain, which comprises several linear epitopes.

Logistic regression as well as ROC analysis confirmed that the ORF2 C-terminus has the highest diagnostic potential of all until now investigated HEV antigens. It is well known that immunogenicity of ORF3 is also determined by the C-terminus [29]. Our data confirm that the N-terminus of ORF3 is not suitable for detection of IgG and IgM antibodies. In contrast the reactivity of the C-terminus correlates very well with the full length ORF3 protein and showed an even better AUC in ROC analysis. A differentiated view on N- and C-terminal parts of ORF3 seems to play an important role not only in serologic diagnostics but also in the newly postulated involvement of ORF3 in viral egress [17,27,30]. For ORF3 the prediction of a transmembrane domain was included in our study to optimize mimicry of naturally occurring antigen conformations and probably explains the predominant seroreactivity of the C-terminal subdomain. Of course this hypothetic secondary structure needs to be confirmed in additional molecular studies. In general the finding of different seroreactivities of morphologic ORF3 subdomains supports the approach to investigate functional ORF1 subdomains separately.

In this study we followed our method for serum profiling experiments [26] achieving an even higher expression level of 60% and a purification efficiency of 100%. As HEV genome size is restricted to about 7,200 nt the complete sequence could be synthesized in vitro thus avoiding the necessity of handling infectious virions during test development and pre-selection of specific viral antigens. In addition the *recom*DOT system applied in this study represents a different and independent assay-format as compared to the already commercially available LIA and ELISA, where the "Mikrogen antigens" are already in use.

Our statistical analysis of genotype 1 ORF2 (O2C vs. O2CGT1) and ORF3 (O3 vs. O3GT1) seroreactivities (IgG and IgM) showed, that respective proteins reacted in the same way with patient serum unrelated to the production method. Correlation coefficients were more than 0.9 between homologous proteins and specificity as well as sensitivity levels appeared to be almost equal. Therefore the described pipeline was proven to be an adequate method to produce proteins of proper quality and characteristics for serum profiling experiments. Furthermore results indicate, that existing commercial HEV assays seem to measure HEV prevalence properly [26].

Germany is a country of low HEV endemicity. Studies could show that genotype 1 infections are commonly associated with traveling in endemic countries and autochthonous genotype 3 infections probably acquired from domestic livestock [31]. As PCR results frequently remain negative and thus no sequence data are obtained genotype during HEV infections often remains unclear. "Serologic genotyping" would easily allow to answer epidemiologic questions when the source of infections is unknown or to estimate the potentially genotype dependent outcome of HEV infections during pregnancy [7]. Comparison of measured signal intensities of genotype 1 and genotype 3 antigens with defined patient sera indicate that there might be a genotype specific reactivity of ORF2 antigens. Further studies with a larger population need to be performed to confirm this finding. Eventually specific sequences of ORF1 also hold the potential for "serologic genotyping" and therefore should be investigated addressing this question.

## Conclusions

This study used epitopes defined by functional domains of all three ORFs and was able to identify novel seroreactive epitopes in each of the three ORFs. Analysis could show that the diagnostic value of identified ORF1 epitopes is not high enough to improve the overall quality of existing test systems. The most potent HEV antigen of the independent systematic screen was found to be the same epitope as the in literature and commercial tests well established C-terminal ORF2 protein. Additionally ORF3 C-terminus is able to compete with antigens used in a commercial test. Summarizing all results obtained from our experiments we conclude that existing commercial HEV assays seem to measure HEV prevalence properly which is based on the high correlation of seroreactivities of homologous antigens. Furthermore we remark that our systematic approach for serum profiling experiments can be used to screen the majority of a complete HEV library for novel antigens. Finally different antigen genotypes could possibly be used to serologically differentiate between genotype 1 and genotype 3 infections and therefore serve as a useful tool to obtain insight into etiological and epidemiological questions of HEV disease.

## Abbreviations

AUC: Area under the curve; ELISA: Enzyme liked immunosorbent assay; Hel: Helicase; HEV: Hepatitis E Virus; LIA: Line-immunoassay; Met: Methyltransferase; MvP-I: Max von Pettenkofer-Institute; ORF: Open reading frame; O2C: C-terminus of ORF2 (MvP-I); O2CGT1: C-terminus of genotype 1 ORF2 (Mikrogen); O2CGT3: C-terminus of genotype 3 ORF2 (Mikrogen); O2NGT1: C-terminus of genotype 1 ORF2 (Mikrogen); O2NGT3: N-terminus of genotype 3 ORF2 (Mikrogen); O2MGT1: Middle part of genotype 1 ORF2 (Mikrogen); O3: ORF3 (MvP-I); O3N: N-terminal part of ORF3 (MvP-I); O3C: C-terminal part of ORF3 (MvP-I); O3GT1: Genotype 1 ORF3 (Mikrogen); O3GT3: Genotype 3 ORF3 (Mikrogen); Plp: Papain like protease; ROC: Receiver operator characteristics; Rdrp: RNA dependent RNA polymerase; V: Variable region; X: X domain; Y: Y-domain.

## Competing interests

AO, MGVP, RH, HN, and AB declare that they have no competing interests. SJ, MS, and UM have received salaries from Mikrogen GmbH. MM is general director of Mikrogen GmbH.

## Authors' contributions

AO performed subcloning of vectors, sequence analysis, establishment and optimization of expression and purification protocols, microarray experiments and statistical analysis as well as writing the manuscript. MGVP optimized expression and purification protocols and participated in drafting the manuscript. RH participated in designing strategies and methods for vector cloning. HN conceived of the study, coordinated the cooperation of both partner institutions and performed PCR analysis and sequencing. SJ designed and performed microarray experiments, carried out data processing and helped to draft the manuscript. MS coordinated and optimized microarray analysis. MM provided basic protocols for microarray analysis and critically revised the manuscript. UM coordinated the cooperation of both partner institutions, designed the study, collected and pre-characterized patient sera, helped with microarray optimization and data interpretation as well as drafting the manuscript. AB conceived of the study, designed bacterial expression vectors and coordinated the cooperation of both partner institutions. All authors read and approved the final manuscript.
